# Proof-of-concept study evaluating humoral primary immunodeficiencies via CJ:KREC ratio and serum BAFF level

**DOI:** 10.1038/s41598-024-64942-4

**Published:** 2024-06-21

**Authors:** Elisa Ochfeld, Amer Khojah, Wilfredo Marin, Gabrielle Morgan, Lauren M. Pachman

**Affiliations:** 1 Pediatric Allergy-Immunology, Ann and Robert H. Lurie Children’s Hospital of Chicago, Chicago, IL USA; 2 Pediatric Allergy-Immunology, Children’s Hospital of Philadelphia, Philadelphia, PA USA; 3 Department of Pediatrics, College of Medicine, Umm Al-Qura University, Al-Abdiyyah campus, Taif road, 21955 Makkah, Saudi Arabia; 4 Division of Pediatric Rheumatology, Ann and Robert H. Lurie Children’s Hospital of Chicago, Chicago, IL USA; 5 Feinberg School of Medicine, Northwestern University, Chicago, IL USA

**Keywords:** Primary immunodeficiency, Antibody deficiency, Common variable immunodeficiency, B cell markers, Humoral primary immunodeficiency, Biomarkers, Diseases, Medical research

## Abstract

Humoral primary immunodeficiencies are the most prevalent form of primary immunodeficiency (PID). Currently, there is no convenient method to quantify newly formed B cells. The aim of this proof-of-concept study was to quantitate the ratio of coding joints (CJs) to Kappa-deleting recombination excision circles (KRECs) and serum B cell activating factor (BAFF) in patients with humoral primary immunodeficiency and assess if they correlate with disease severity. This IRB-approved study was conducted at one academic children’s hospital. Patients with humoral PIDs and healthy controls were included. CJ and KREC levels were measured via qPCR. Serum BAFF levels were measured using Mesoscale. 16 patients with humoral PID and 5 healthy controls were included. The mean CJ:KREC ratio in the CVID, antibody deficiency syndromes, and controls groups, respectively were 13.04 ± 9.5, 5.25 ± 4.1, and 4.38 ± 2.5 (*p* = 0.059). The mean serum BAFF levels in CVID, antibody deficiency syndromes and controls were 216.3 ± 290 pg/mL, 107.9 ± 94 pg/mL and 50.9 ± 12 pg/mL, respectively (*p* = 0.271). When the CVID patients were subdivided into CVID with or without lymphoproliferative features, the BAFF level was substantially higher in the CVID with lymphoproliferation cohort (mean 372.4 ± 361 pg/mL, *p* = 0.031). Elevated CJ:KREC ratio was observed in CVID, although statistical significance was not achieved, likely due to the small sample size. Serum BAFF levels were significantly higher in CVID patients with lymphoproliferative features. We speculate that the CJ:KREC ratio and serum BAFF levels can be utilized in patients with humoral PID, once more extensive studies confirm this exploratory investigation.

## Introduction

Primary immunodeficiency diseases (PIDs), also referred to as Inborn Errors of Immunity (IEI), are distinct disorders caused by inherited defects in the immune system which predispose patients to frequent and severe infections, increase risk of autoimmunity and immune dysregulation, and can be life-threatening. B cell (humoral) defects involving predominantly antibody deficiencies are the most common form of primary immunodeficiency, accounting for approximately half of all PIDs^1,2^. At this time, many humoral PIDs are diagnosed based on history and laboratory abnormalities. Laboratory evaluation often includes lymphocyte subset quantification, naïve and memory B cell phenotyping, immunoglobulin levels, and functional studies including vaccine response to immunization and serum specific antibody titers^3^. While B cell subset phenotyping can quantify the number of naïve, memory, and transitional B cells, and can be helpful in the diagnosis and prognosis of humoral PID^4^, this test is lacking in ability to quantify newly formed B cells (*i.e.* not only naïve B cells but recently formed B cells, produced during expansion and proliferation: a measure of new B cell output). Transitional B cell markers can be used to identify this population to some degree, however B cell subset phenotyping is expensive, and the values rapidly fluctuate. Currently, there is not a clinically useful way to reliably measure the number of newly formed B cells. This measurement could be exceptionally useful in the evaluation and management of humoral PID.

The formation of new B cells requires B cell receptor (BCR) V(D)J recombination and involves production of a signal joint, or kappa-deleting recombination excision circle (KREC). KRECs are circular excision products that do not replicate during B cell division. The KREC remains only in one B cell and not in that cell’s progeny^5^. The site of DNA recombination after KREC excision is termed the coding joint (CJ), and contrastingly, is found in all subsequent B cell progeny^5^. The ratio of CJ to KREC DNA can be determined via real-time quantitative PCR (qPCR). This ratio can be utilized to estimate the number of B cell divisions that have occurred in a B cell population, and to measure the number of newly formed B cells^5^. This could be used as a clinical marker to assess the severity of B cell immunodeficiencies, and possibly help predict the risk of disease complications, including autoimmunity, lymphoproliferation or susceptibility to infection. An elevated CJ:KREC DNA ratio can also indicate oligoclonal B cell expansion, a hallmark of B cell immune response in both autoimmunity and acute infection.

B Cell Activating Factor (BAFF), a cytokine produced by activated macrophages and dendritic cells, promotes B cell proliferation and survival. BAFF level increases can drive B cell expansion and B cell hyperplasia. BAFF levels are known to be elevated in patients with autoimmune disease^6,7^. Elevated BAFF levels are associated with the recurrence of diseases like systemic lupus erythematosus (SLE) and Sjogren’s syndrome, particularly after undergoing Rituximab therapy.^8,9^ Rituximab is a monoclonal antibody that targets CD20 and is frequently used to treat autoimmune diseases. This therapy typically leads to the depletion of peripheral B cells and, in certain instances, triggers prolonged hypogammaglobulinemia^10^. Furthermore, BAFF levels have been implicated in the pathophysiology of humoral primary immunodeficiency disease^11,12^. While BAFF levels are elevated in CVID, one study did not find an association between BAFF and autoimmunity in CVID patients, though this may have been limited by the small sample size of patients with autoimmunity in this cohort^12^. Further studies are necessary to elucidate these relationships.

The primary aim of this proof-of-concept study was to quantify CJ and KREC DNA in patients with humoral primary immunodeficiency, and to evaluate B cell output in order to assess this ratio as a potential indicator of disease severity. Our hypothesis is that the ratio of CJ: KREC DNA in patients with humoral primary immunodeficiency will be elevated compared to healthy controls, and that the CJ:KREC ratio will be highest in those with more severe forms of humoral primary immunodeficiency, such as common variable immunodeficiency (CVID), compared to those with less severe forms– including antibody deficiency syndromes. We hypothesized that serum BAFF levels would also be higher in those with more severe humoral PIDs compared to those with less severe humoral PIDs, and lowest in healthy controls.

## Methods

This IRB-approved study (IRB 2018-1737) was conducted at Ann & Robert H. Lurie Children's Hospital of Chicago. Patients with humoral primary immunodeficiencies (including hypogammaglobulinemia, specific antibody deficiency, or CVID) aged 6 months to 22 years were eligible for inclusion in the study. CVID diagnosis was based on the 1999 proposal established by the European Society for Immunodeficiencies and the Pan American Group for Immunodeficiency (Supplemental Table 1)^13^. Specific antibody deficiency was defined as patients with recurrent sinopulmonary infections and insufficient vaccine responses, though they did not fulfill the diagnostic criteria for CVID. Hypogammaglobulinemia within the study included patients presenting with recurrent infections and serum IgG levels 2 standard deviations below the age-specific mean but did not fulfill the CVID diagnostic criteria. Of note, a subset of these patients exhibited chromosomal or genetic disorders (Supplemental Table 2). Patients suspected of having transient hypogammaglobulinemia of infancy were deliberately excluded from the study. We also excluded any patients who had active infections or were experiencing flare-ups of autoimmune diseases at the time of blood collection. This precaution was taken to prevent potential confounding of the study results. Data was obtained from the electronic medical record, including sex, age, race, PID diagnosis, clinical manifestations including infections and autoimmune disease, and immune laboratory evaluations.

**Table 1:** Patient Characteristics (*n* = 16).

Characteristic (*n*, %)	Antibody deficiency syndromes (*n* = 8)	CVID (*n* = 8)	Overall (*n* = 16)
Age (Mean, ± SD)	6.67 ± 4.77	14.63 ± 6.14	10.65 ± 6.72
Sex			
Female	2 (25%)	2 (25%)	4 (25%)
Male	6 (75%)	6 (75%)	12 (75%)
Race/Ethnicity			
White	4 (50%)	7 (87.5%)	11 (68.75%)
Asian	1 (12.5%)	0 (0%)	1 (6.25%)
Black	0 (0%)	0 (0%)	0 (0%)
Hispanic	3 (37.5%)	1 (12.5%)	4 (25%)
Autoimmunity	0 (0%)	4 (50%)	5 (31.25%)
Interstitial lung Disease	0 (0%)	2 (25%)	2 (12.5%)
Splenomegaly	0 (0%)	3 (37.5%)	3 (18.75%)
Cytopenias	0 (0%)	5 (62.5%)	5 (31.25%)
Receiving IG Replacement	5 (62.5%)	7 (87.5%)	12 (75%)

Coding joint and KREC DNA levels were measured via real-time quantitative PCR from DNA extracted from peripheral blood mononuclear cells (PBMCs), harvested with an arm to freezer time of under 2 h and a 94% viability. This study protocol was adapted from prior research from van Zelm, et al.^5^. Real-time quantitative PCR assays for the detection of coding joints and signal joints were utilized^5^. Primers and probes amplified the intron RSS-Kde rearrangements (coding joints) and signal joints (KRECs). The qPCR protocol contained 12.5 µL of TaqMan Universal MasterMix (Applied Biosystems), 2 µL of sample DNA, 2.5 µL of forward primer, 2.5 µL of reverse primer, 1 µL of FAM-TAMRA labeled probe, and 4.5 µL of DI RNAse/ DNAse free water. The total volume per well was 25 µL. PCR plates were run on an Applied Biosystems QuantStudio 6 Flex Real-Time PCR System. PBMC DNA extraction was performed using a standardized protocol. For each sample, C_T_ values of coding joint and signal joint were compared. Per prior work by van Zelm^5^, the ΔC_T_ (C_T KREC_–C_T CODING JOINT_) represents the mean number of B cell divisions^5^. Additionally, utilizing the formula ^2^logΔCT, van Zelm demonstrated that this equates to the coding to signal joint ratio (CJ: KREC ratio), which can be used to measure the in vivo replication history of a B cell subset, and is, therefore, a marker of B cell expansion. This approach has been validated with in vitro proliferation studies^5^. Serum soluble BAFF level (pg/mL) was measured using Mesoscale technology. A similar analysis was performed in healthy pediatric controls (using de-identified samples) (Supplemental Table 3). Software utilized to perform the statistical analysis included PRISM and SPSS.

### Ethical approval and consent to participate

The study was conducted according to the guidelines of the Declaration of Helsinki and approved by the Institutional Review Board of Ann & Robert H. Lurie Children's Hospital of Chicago (IRB 2018-1737). All experimental protocols were approved by the Ann & Robert H. Lurie Children’s Hospital institutional review boards. All of the participants and/or their legal guardian(s) signed written informed consent/assent.

## Results

**Figure 1:**
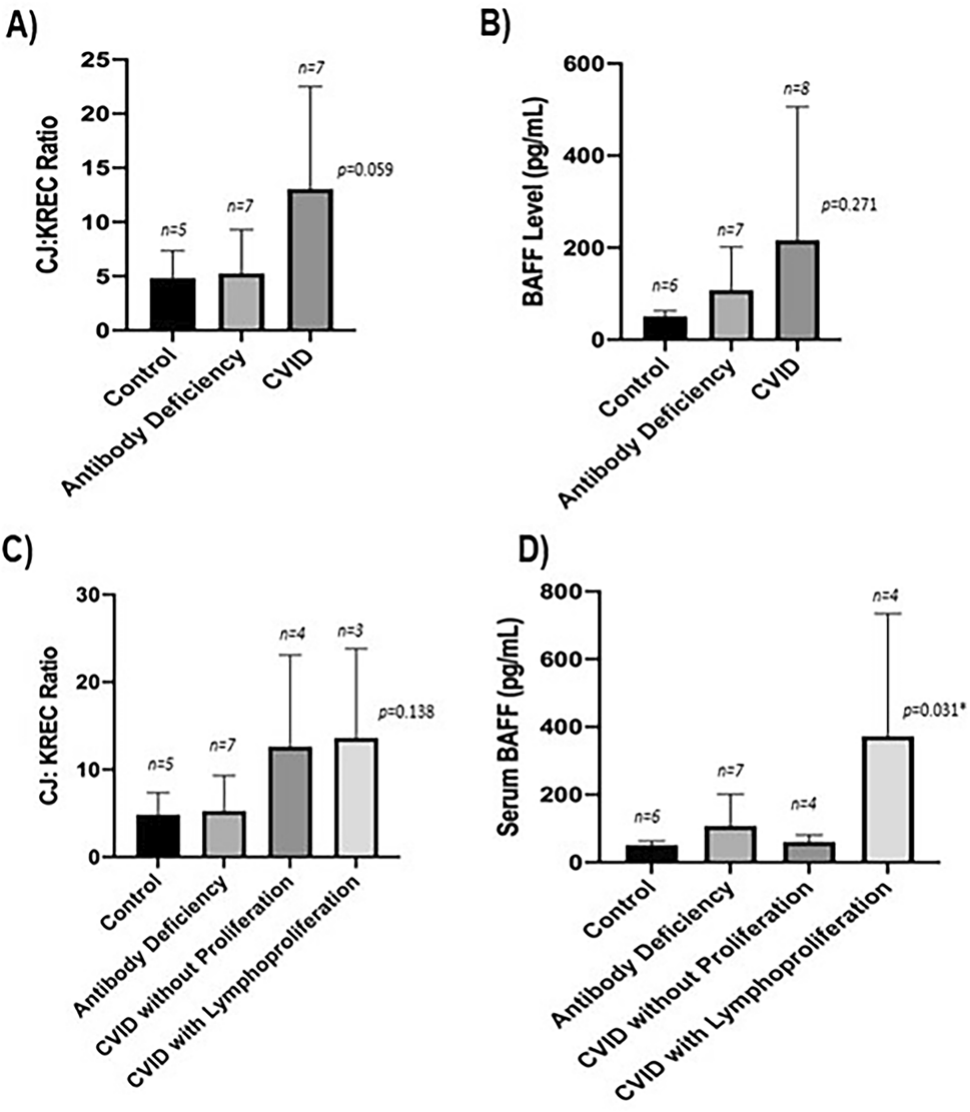
Mean CJ: KREC ratio and serum BAFF levels in patients with humoral immunodeficiency and in healthy controls. (**A**) Mean CJ:KREC ratio in the CVID, antibody deficiency syndromes, and controls were, 13.04, 5.25, 4.38 respectively (*p* = 0.059) (one way ANOVA, *p* = 0.059) (**B**) Mean serum BAFF in CVID, antibody deficiency syndromes, and controls 216.3, 107.9 and 50.9, respectively (one way ANOVA *p* = 0.271) (**C**, **D**) CVID patients were subdivided into CVID with or without lymphoproliferative features, The CJ:KREC ratio was similar between the CVID subgroups (mean of 12.62 vs. 13.61). However, the BAFF level was substantially higher in the CVID with lymphoproliferation cohort (mean 372.4 pg/mL and *p* = 0.031).

Sixteen patients with humoral primary immunodeficiencies were included in the analysis. Demographic characteristics are reported in Table 1 and supplemental Table 2. The mean age of patients was 10.66 ± 6.7 years old. Patients were divided by their humoral PID diagnosis into two groups, either antibody deficiency syndrome (including specific antibody deficiency and/or hypogammaglobulinemia) or CVID. The mean CJ:KREC ratio in the CVID, antibody deficiency syndromes, and controls were, PID cohort was 13.04 ± 9.5, 5.25 ± 4.1, and 4.38 ± 2.5, respectively (Fig. 1A). The statistical significance was not achieved, likely due to the small sample size, but did approach significance (*p* = 0.059). Mean serum BAFF in CVID, antibody deficiency syndromes, and controls 216.3 ± 290 pg/mL, 107.9 ± 94 pg/mL and 50.9 ± 12 pg/mL, respectively (Fig. 1B, *p* = 0.271).

**Figure 2:**
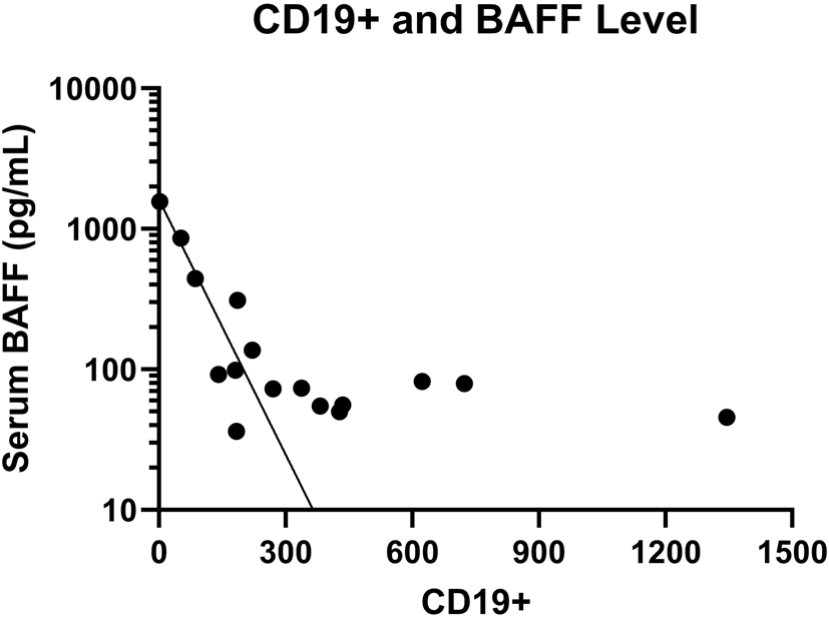
Mean serum BAFF level inversely and logarithmically correlated with CD19 + B cell count (R^2^ = 0.96) in patients with humoral immunodeficiency.

CVID patients were subdivided into CVID without lymphoproliferative features (patients with primarily infectious complications) and CVID with lymphoproliferative features (including splenomegaly, interstitial lung disease, autoimmunity, and cytopenia). While the CJ:KREC ratio was strikingly similar between the CVID subgroups (mean of 12.62 ± 10.5 vs. 13.61 ± 10.2, Fig. 1C), the BAFF level was substantially higher in the CVID with lymphoproliferation cohort (mean of 372.4 ± 361 pg/mLvs 60.15 ± 20.9 pg/mL, Fig. 1D), and when compared across all groups this achieved statistical significance (*p* = 0.031),

Assessing these immunologic markers compared to routinely clinically utilized immunologic laboratory markers, we found that serum BAFF level inversely and logarithmically was correlated with CD19 + B cell count (R^2^ = 0.96, Fig. 2). However, the correlation between the between CJ:KREC and BAFF levels was not significant (r = 0.36, *p* = 0.2) likely due to the small sample size.

## Discussion

**Figure 3:**
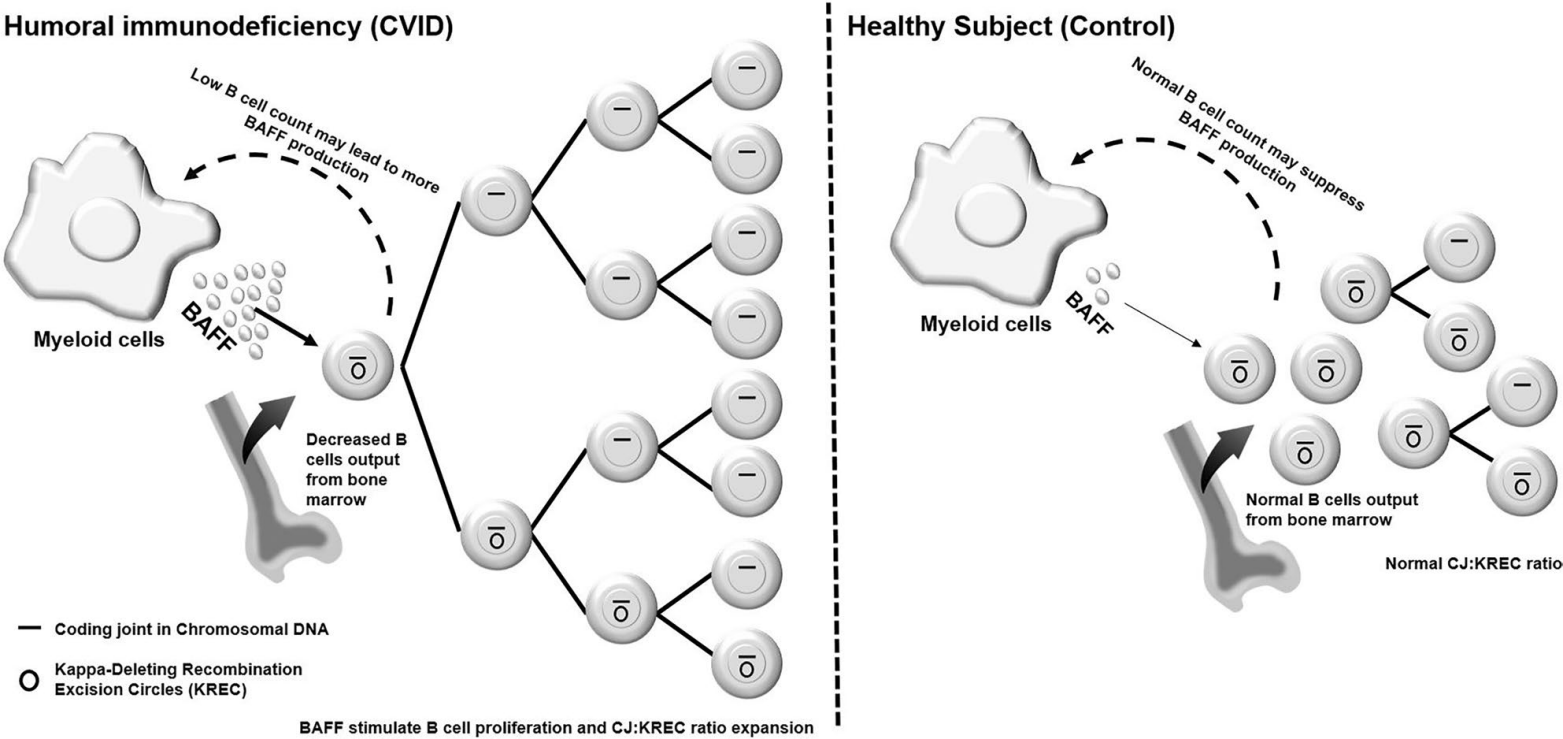
Study hypothesis diagram. Patients with humoral primary immunodeficiency such as CVID and to less degree antibody deficiency syndromes have increased CJ:KREC ratios and elevated serum BAFF levels. We hypothesize that patients with CVID have reduced naïve B cell output from the bone marrow, potentially leading to increased serum BAFF production by myeloid cells. This, in turn, may stimulate B cell division and accelerate the expansion of the CJ:KREC ratio. The diagram on the left illustrates the anticipated changes in CVID patients compared to healthy individuals (panel on the right).

The CJ:KREC ratio and serum BAFF can evaluate new B cell output and can be utilized as a marker of disease severity in humoral primary immunodeficiency (Fig. 3). The severity and prognosis of humoral PIDs can be estimated and the trend visualized over time using the CJ:KREC ratio and serum BAFF level. Patients with more severe forms of humoral primary immunodeficiency such as CVID have increased CJ:KREC ratios and increased serum BAFF levels, compared to those with less severe humoral PIDs such as antibody deficiency syndromes. Of note, a lower CD19 + B cell count was correlated inverse-logarithmically with a higher serum BAFF level in our study. This is consistent with prior research which showed that X- linked agammaglobulinemia (XLA) patients have significantly higher soluble BAFF concentrations compared to healthy controls (*p* < 0.001)^14^. The current concepts of the pathophysiology of serologic immunodeficiency substantiates our findings that the more significant the inherited B cell deficiency and the lower the B cell count, the higher the resulting serum BAFF level. BAFF is attempting to stimulate production of B cells, and without the negative feedback from resultant increased B cell production, BAFF levels stay elevated and may continue to rise. This, in theory, could lower B cell tolerance and allow for autoimmunity and lymphoproliferation. While BAFF correlated inversely with B cell count, CJ:KREC ratio trended towards correlating inversely with switched memory B cell percentage, as these cells typically undergo multiple rounds of cell division. A higher CJ:KREC ratio was associated in our study with more severe humoral immunodeficiency (CVID). CVID patients with increased autoimmunity, granulomatous disease and lymphoproliferation have reduced switched memory B cells compared to CVID patients with a milder phenotype (primarily infectious complications)^3,15,16^. It's important to note that the oligoclonal B cell expansion indicated by an elevated CJ:KREC ratio could potentially arise from various factors such as infections, autoimmunity, or malignancy. Therefore, in clinical practice, considering additional tests that can differentiate among these possible diagnoses would provide valuable insights into the underlying mechanisms contributing to the observed elevated CJ:KREC ratios. For example, a complete blood count (CBC) could prove beneficial in identifying cytopenia, which is the most common autoimmune manifestation in CVID^17^, or leukocytosis, which may suggest the presence of acute infection^18^. Furthermore, in the appropriate clinical contexts, advanced flow cytometry, bone marrow biopsy, and imaging studies may be necessary to rule out the possibility of leukemia and lymphoma^19^.

Regarding the pathophysiology underlying PID-associated lymphoproliferation, substantial research has focused on interstitial lung disease (ILD). ILD is associated with autoimmune cytopenias and splenomegaly in CVID patients, highlighting the systemic immune dysregulation underlying these processes^11,20^. BAFF-related B cell hyperplasia is known to drive ILD in CVID^21^. BAFF levels were elevated in the serum of patients with CVID that had progressive ILD, but not in those with stable ILD, suggesting BAFF is linked to interstitial lung disease activity^21^. In the two patients in our study with very elevated BAFF levels, these patients all had a history of substantial lung disease. Further investigation into the interplay between BAFF and ILD is warranted, and this extends beyond PID, involving rheumatologic pathophysiology as well. It has been demonstrated that in Juvenile Dermatomyositis (JDM), patients with rapidly progressive interstitial lung disease displayed markedly elevated levels of BAFF and APRIL^22^. JDM patients with evidence of oligoclonal B cell expansion prior to Rituximab therapy (as demonstrated by an elevated CJ:KREC ratio) had a more favorable clinical response to Rituximab, and these patients also showed an increase in serum BAFF levels after Rituximab^23^. BAFF-driven splenomegaly has been studied primarily in the context of infectious disease. BAFF deficiency suppresses splenomegaly in mice during *Leishmania donovani* infection^24^, and BAFF mRNA levels in the spleen of patients infected with *Plasmodium falciparum* were significantly elevated compared to normal spleens^25^.

Importantly, qPCR quantification of CJ and KREC DNA could replace more expensive laboratory tests such as flow cytometric evaluation of B cells and B cell subsets, especially in international and/or limited health-resource settings where flow cytometry may not be available. PCR is significantly more cost-effective compared to flow cytometry. Additionally, PCR can be performed using very small quantities of blood, as utilized in TREC evaluation in newborn screening (NBS) for Severe Combined Immunodeficiency (SCID) via dried bloodspot. Further clinical implications for the CJ:KREC ratio in comparison with serum BAFF level include: to evaluate response to B cell depleting therapy^23^; to help predict response to immunomodulators and guide therapeutic management, and, finally, to monitor B cell reconstitution after hematopoietic stem cell transplantation. Further research is required in each of these areas.

From this proof-of-concept study, we reason that (1) CJ:KREC ratio can be used to assess the severity of humoral PID and aid in differentiation between antibody deficiency syndromes and CVID, and (2) that the serum BAFF level can supplement the CJ:KREC ratio data to evaluate within humoral PID for lymphoproliferative features. The CJ:KREC ratio may be especially helpful in circumstances where a full immunologic evaluation cannot be completed. For example, a patient with a history of hypogammaglobulinemia, receiving immunoglobulin replacement therapy, who develops low IgA or IgM, and cannot have vaccine antibody responses re-evaluated. Additionally, the CJ:KREC ratio and BAFF level could be performed at initial immunologic evaluation in patients with humoral PIDs, and then serially tested over time, to monitor for disease progression and signal the occurrence of new lymphoproliferation.

There are limitations to this study: Firstly, the sample size was small, underscoring the need for validation in larger-scale studies to ensure the robustness and generalizability of our findings. Additionally, expanding future studies to include adults with primary immunodeficiencies (PIDs) or patients with secondary antibody deficiencies would be valuable, particularly given the limited availability of biomarkers in these patient populations. Furthermore, This was a single-center study, which can impact generalizability as well. Finally, we did not serially evaluate CJ:KREC ratio or BAFF level, but this is an area of potential future research. We speculate that the data acquired from this testing will prove to be useful in understanding the humoral immune response.

### Supplementary Information

Supplementary Information.

## Data Availability

The data that support the findings of this study are available from the corresponding author upon reasonable request.

## Abbreviations

CJ: Coding joint
KREC: Kappa-deleting recombination excision circle
BAFF: B cell activating factor
PID: Primary immunodeficiency
CVID: Common variable immunodeficiency
IEI: Inborn error of immunity
qPCR: Quantitative polymerase chain reaction
SLE: Systemic lupus erythematosus
XLA: X linked agammaglobulinemia
ILD: Interstitial lung disease
APRIL: A proliferation-inducing ligand
JDM: Juvenile dermatomyositis
NBS: Newborn screening
SCID: Severe combined immunodeficiency
