# Supplementary material for: Proof-of-concept study evaluating humoral primary immunodeficiencies via CJ:KREC ratio and serum BAFF level
Source: Sci Rep. 2024 Jun 21;14:14356. doi: 10.1038/s41598-024-64942-4 (PMC11192915; doi:10.1038/s41598-024-64942-4)
Supplement: Supplementary file 1 — Supplementary Information. [file 41598_2024_64942_MOESM1_ESM.docx]

Supplemental Table 1: CVID diagnostic criteria.

| The patient must meet all of the following criteria for the diagnosis of CVID: |
| --- |
| 1. Low serum IgG level (at least 2 SD below the mean for age) in addition to low serum IgA or IgM levels (at least 2 SD below the mean for age) |
| 1. Age of diagnosis greater than 2 years of age |
| 1. Poor vaccines response and/or absent isohemagglutinins |
| 1. Exclude other causes of hypogammaglobulinemia such as drug-induced hypogammaglobulinemia, HIV, and malignancy |

Supplemental Table 2: Genetic data of the study subjects

| ID | Sex | Race | Age (Years) | Diagnosis | IVIG | Cytopenia | Lung disease | Autoimmunity | Splenomegaly | genetic testing | CJ: KREC ratio | and serum BAFF |
| --- | --- | --- | --- | --- | --- | --- | --- | --- | --- | --- | --- | --- |
| ID19 | M | H | 10 | SAD | Yes | No | No | No | No | 2q14.3 duplication | 3.2 |  |
| ID7 | M | W | 9 | SAD | No | No | No | No | No | Not done | 1.16 | 55.8 |
| ID9 | M | H | 12 | HYPO | Yes | No | No | No | No | Not done | 2.5 | 54.7 |
| ID4 | M | H | 8 | HYPO | Yes | No | Yes^a^ | No | No | deletion on 22q12.3-q13.1 and small duplication chromosome 19 | 6.1 | 309 |
| ID5 | M | A | 1 | SAD | Yes^*^ | No | No | No | No | Angelman syndrome |  | 45 |
| ID15 | F | W | 2 | SAD | No | No | No | No | No | TNFR5F13B gene heterozygous mutation c.542C>A | 5.1 | 79 |
| ID16 | M | W | 1 | SAD | No | No | No | No | No | ATM (C.5228C>T and C.8282dup) | 13.62 | 137 |
| ID6 | F | W | 11 | SAD | Yes | No | No | No | No | heterozygous variants of CFTR | 4.94 | 73.5 |
| ID12 | M | H | 10 | CVID | Yes | Yes (N) | No | No | No | homozygous for variant in EFTUD1 (c.26457>A) | 26.9 | 72.6 |
| ID13 | M | W | 17 | CVID | No | Yes (N) | No | No | No | Negative | 2.1 | 36 |
| ID14 | M | W | 5 | CVID | Yes | No | No | No | No | heterozygous TNFR5F13B variant P.A181E | 12.5 | 49 |
| ID10 | M | W | 9 | CVID | Yes | No | No | No | No | negative | 8.8 | 82 |
| ID8 | M | W | 23 | CVID+ | Yes | Yes (HA) (ITP) | No | Yes | Yes | Not done | 25.33 | 92.1 |
| ID11 | M | W | 20 | CVID+ | Yes | Yes (ITP) | Yes | Yes | Yes | Not done | 6.7 | 442.5 |
| ID17 | F | W | 20 | CVID+ | Yes | No | No | Yes | No | Not done | 8.7 | 98.8 |
| ID18 | F | W | 14 | CVID+ | Yes | Yes (ITP) | Yes | Yes | Yes | RAB27 genetic variant |  | 856.3 |

*= only few doses, ^a^= lung disease secondary to heart disease

F= Female, M= Male, W= White, H= Hispanic, A= Asian, SAD= Specific antibody deficiency, Hypo= Hypogammaglobulinemia, CVID= Common variable immune deficiency, CVID+= Common variable immune deficiency with lymphoproliferative features (including splenomegaly, interstitial lung disease, and/or autoimmunity), N= Neutropenia, HA= Hemolytic Anemia, ITP= Idiopathic thrombocytopenic purpura

Supplemental Table 3: CJ:KREC ratio in healthy controls

| ID | Sex | Age range | CJ:KREC ratio |
| --- | --- | --- | --- |
| 1 | Female | 5-9 years | 6.9 |
| 2 | Male | 10-14 years | 5.2 |
| 3 | Female | 5-9 years | 2.5 |
| 4 | Male | 15-18 years | 2.0 |
| 5 | Female | 10-14 years | 7.6 |
